# Total Hip Replacement: Psychometric Validation of the Italian Version of Forgotten Joint Score (FJS-12)

**DOI:** 10.3390/jcm12041525

**Published:** 2023-02-15

**Authors:** Umile Giuseppe Longo, Sergio De Salvatore, Giulia Santamaria, Anna Indiveri, Ilaria Piergentili, Giuseppe Salvatore, Maria Grazia De Marinis, Benedetta Bandini, Vincenzo Denaro

**Affiliations:** 1 Research Unit of Orthopaedic and Trauma Surgery, Fondazione Policlinico Universitario Campus Bio-Medico, Via Alvaro del Portillo, 00128 Roma, Italy; 2 Research Unit of Orthopaedic and Trauma Surgery, Department of Medicine and Surgery, Università Campus Bio-Medico di Roma, Via Alvaro del Portillo, 00128 Roma, Italy; 3 Research Unit Nursing Science, Campus Bio-Medico di Roma University, 00128 Rome, Italy

**Keywords:** Forgotten Joint Score, italian, replacement, total hip arthroplasty, total hip replacement, validation

## Abstract

Background: One million Total Hip Replacements (THA) are thought to be performed annually. To measure prosthesis awareness throughout daily activities, the FJS-12 patient-reported outcome scale was developed. This article’s goal is to undertake a psychometric validation of the Italian FJS-12 among a sample of related THA patients. Methods: Between January and July 2019, data from 44 patients were retrieved. The participants were required to complete the Italian version of FJS-12 and of the WOMAC at preoperative follow-up, after two weeks, 1, 3, and 6 months postoperatively. Results: The Pearson correlation coefficient between the FJS-12 and WOMAC was 0.287 (*p* = 0.002) at preoperative follow-up, r = 0.702 (*p* < 0.001) at 1 month, r = 0.516 (*p* < 0.001) at 3 months and r = 0.585 (*p* < 0.001) at 6 months. The ceiling effect surpassed the acceptable range (15%) for FJS-12 in 1 month (25.5%) and WOMAC in 6 months follow-up (27.3%). Conclusions: The psychometric validation of the Italian version of this score for THA was executed with acceptable results. FJS-12 and WOMAC reported no ceiling and floor effects. Therefore, to distinguish between patients who had good or exceptional results following UKA, the FJS-12 could be a reliable score. Under the first four months, FJS-12 had a smaller ceiling effect than WOMAC. It is recommended to use this score in clinical research concerning the outcomes of THA.

## 1. Introduction

Around the world, one million total hip arthroplasty (THA) surgeries are thought to be performed annually [1,2]. Between 2014 and 2017, there were 370.000 THAs carried out in the United States, while Italy conducted 91.428 THAs in 2014, representing a 44.0% increase from 2001 to 2014. [3,4]. The incidence of THA has been increasing annually, according to reports from throughout the world [5], mostly as a result of population aging [6]. With the development of new technologies and implants, the patient’s expectations and the necessity to improve outcomes increase. Thereby, it is mandatory to develop new systems to assess outcomes after THA to match the patient’s expectations [7]. Nowadays, Patient-Reported Outcome Measurements (PROMs) are widely employed to evaluate the living quality of patients with THA [8,9,10]. The Western Ontario and McMaster Universities Osteoarthritis Index (WOMAC) is one of several approved scores that evaluates results following THA [11]. WOMAC has been found to have a moderate ceiling effect [12], raising some doubts about its ability to discern between good and exceptional outcomes despite its great reliability and repeatability [13]. Due to the improvements in joint replacement techniques, patients’ expectations have increased, requiring more satisfactory outcomes. Furthermore, while having a longer life expectancy, active implant patients still need to have revision surgery because of a variety of implant failure-related factors [14]. With improved patient outcomes, it is mandatory to develop new PROMs, with a more targeted question and discriminatory power [15].

One good score to use after THA is the Forgotten Joint Score-12 (FJS-12). The FJS-12 is a patient-reported outcome scale created to measure prosthesis perception throughout daily tasks [16,17]. It measures the level of perception of the prosthetic joint with a low ceiling effect and was created by Behrend et al. in 2012 [16]. The validity of this questionnaire in its numerous language translations has been shown in several articles [18].

In the current literature, no other study evaluates the Italian FJS-12 for THA patients, highlighting an overall need for further research. Furthermore, translating this PROM into different languages is fundamental to using this tool worldwide and developing it among countries since linguistic comprehension could represent a limitation in clinical practice.

This article’s goal is to undertake a psychometric validation of the Italian FJS-12′s among a sample of related THA patients.

## 2. Materials and Methods

### 2.1. Validation Study

Prospective, observational research was carried out to assess the responsiveness, and reliability of the Italian FJS-12 for THA. The study involved 55 patients that received THA from January to July 2019.

**Figure 1:**
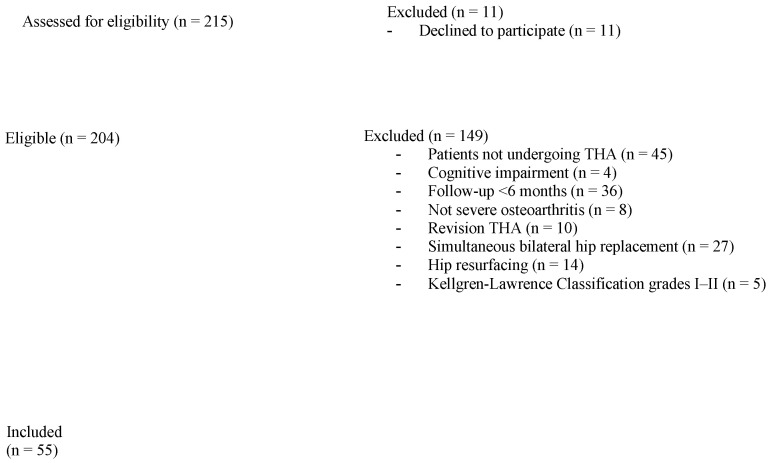
Exclusion process.

Severe hip osteoarthritis (Kellgren-Lawrence Classification Grade III–IV) [19], persistent pain, hip replacement surgery, and a minimum of six months of follow-up following surgery were the inclusion criteria. The same surgical implants were used on all patients who received THA. The prostheses implanted in this study were Avenir^®^ and Fitmore^®^ (both ZimmerBiomet Inc., Warsaw, IN, USA) and MasterLoc^®^ and SMS^®^ (both Medacta International, Castel San Pietro, Switzerland) selected and implanted according to the individual necessities of each patient. Patients with cognitive impairment, simultaneous bilateral hip replacement, hip resurfacing, revision surgery, and Kellgren-Lawrence Classification Grades I-II were all excluded from the research (Figure 1).

The Italian versions of the WOMAC and of the FJS-12 were completed by each patient at preoperative follow-up, and postoperatively at two weeks, one month, three months, and six months. In order to conduct the test-retest, the patients also completed the pre-operative WOMAC and FJS-12 questionnaires twice, two weeks apart, before surgery. Prior to data collection, the research was authorized by the Institutional Review Board (IRB). 

### 2.2. Assessment Instruments

#### 2.2.1. FJS-12

The FJS-12 is a PROM created to measure prosthesis perception throughout simple tasks [16]. The FJS-12 comprises twelve questions with a Likert response format ranging from one to five. Each patient’s item scores, starting from a minimum of 12 up to a maximum of 60, are summed. The outcomes are rated between 0 and 100 (high scores identify positive results). Comparing the FJS-12 to other clinical scores, it has sufficient discriminating ability and a limited ceiling impact [16]. The Italian FJS-12 was used [20,20].

#### 2.2.2. WOMAC

The WOMAC is a self-reported multidimensional questionnaire used to assess stiffness, pain and disability. It comprises 24 questions on a 0 to 4 Likert response structure, including 17 questions about function and 17 questions for pain and stiffness. The summarized scores start from 0 up to 96. The results can be standardized between 0 and 100, where 100 represents the highest functioning status level. In addition, according to the COnsensus-based Standards for the selection of health Measurement INstruments (COSMIN), the study by Gandek et al. demonstrated the strong reliability and internal consistency of this score (Cronbach’s > 0.90) [21]. Consequently, this study chose it as the comparison survey for these reasons. 

### 2.3. Reliability

To rate the reliability, measurement error, internal consistency, and test-retest reliability were identified [22]. The internal consistency was calculated with Cronbach’s α, with a value greater than 0.7 identified as sufficient [23]. 

As per the COSMIN protocol, the retest was concluded two weeks after the first assessment [22]. Therefore, the test-retest reliability was computed with the Intraclass Correlation Coefficient (ICC) between the scores assessed preoperatively and two weeks postoperatively.

The measurement error [22] was computed by the Standard Error of Measurement (SEM) and the Minimal Detectable Change (MDC). 

The SEM was computed using the formula SD × √(1 − α) (SD = Standard Deviation and α = Cronbach’s α). The MDC was computed using the formula SEM × 1.96 × √2. The minimal personal change in a value that may be considered to be a substantial shift is known as the MDC. To evaluate the validity, the Pearson correlation coefficient (r) between FJS-12 and WOMAC at all times was computed [22]. Values of r ≥ 0.3 correlate were supposed.

Effect size (ES) and MDC were computed to evaluate the responsiveness [22]. ES was computed by comparing the score at study inception to the scores at 1, 3, and 6 months after surgery, as well as the scores at 1 and 3 and 6 months. Finally, it has been computed between the 3 and 6 month scores. ES was computed as the ratio of mean difference and SD (Cohen’s d). Also, if MDC was lower than the Minimal Important Change (MIC), a positive evaluation or responsiveness is supposed (MIC = 0.5 × SD).

### 2.4. Statistical Analysis

A priori power analysis with ES of 0.6 for FJS-12 from preoperative to final follow-up as in literature [1], sig. level = 0.05 and power = 0.80 have evaluated a cohort of at least 24 participants. Descriptive statistics are reported as means and Standard Deviations (SD). 

The rates for patients receiving the greatest (100) and lowest (0) FJS-12 scores, respectively, are referred to as ceiling effects and floor effects, respectively. Less than 15% of the ceiling and floor effects were regarded as adequate.

Statistical analysis was carried out with SPSS 26.0 and power analysis with G*Power 3.1.9.4.

## 3. Results

Between January to July 2019, 55 patients (26 female, 29 male; mean age of 74 ± 11) were enrolled in the research. 

The mean value of FJS-12 at inception was 35.08 ± 15.32; 1-month postoperatively was 81.33 ± 18.99; 3-months postoperatively was 80.99 ± 13.42 and at final follow-up was 80.60 ± 15.83 (Table 1). The mean score of WOMAC is reported in Table 1.

Cronbach’s α was assessed at every follow-up. A range of Cronbach’s α from 0.673 to 0.922 identified sufficient internal consistency for the FJS-12 (Table 1).

The test-retest reliability was sufficient in every case, with an ICC of 0.987 (CI: 0.977, 0.992; *p* < 0.001). 

The SEM at baseline FJS-12 was 8.760, while MDC was 24.281. The SEM at the final follow-up FJS-12 was 6.563, and the MDC was 18.192 (Table 2).

The Pearson correlation coefficient between FJS-12 and WOMAC was 0.287 (*p* = 0.002) preoperatively, r = 0.702 at 1 month, r = 0.516 at 3 months, and r = 0.585 at 6 months (Table 3). 

Construct validity revealed a high-moderate correlation between the two surveys, with the exception of preoperative follow-up. At every follow-up, the results were evaluated with pairwise comparisons in order to gauge the FJS-12′s reactivity over time (Table 4). The same assessment was conducted for WOMAC (Table 5). 

The mean difference between preoperative and follow-up at 1 month was −46.25, with a great ES (Cohen’s d = 2.534, *p* < 0.001). The WOMAC also showed a great ES (Cohen’s d = 2.085, *p* < 0.001), with a mean difference of −41.818. 

Finally, the MDC was always greater than the respective MIC; for this reason, no positive evaluation for responsiveness could be given.

The floor effect was 0% for FJS-12 and WOMAC at every follow-up.

The ceiling effect was 0% for FJS-12 preoperatively and WOMAC preoperatively and 1 month after the procedure. The ceiling effect was less than the acceptable range (15%) for FJS-12 at 3 and 6 months (9.1% and 12.7%) and WOMAC at 3 months postoperatively (5.5%). The ceiling effect was greater than the acceptable range (15%) for FJS-12 at 1 month (25.5%) and WOMAC at final follow-up (27.3%) (Table 6).

## 4. Discussion

This study performed the psychometric validation of the Italian FJS-12 for THA, resulting in a good test-retest reliability and a mild association with the WOMAC.

The absence of pain and an adequate Range Of Motion (ROM), are the first steps for the patient to accept the hip prosthesis [24]. Therefore, the FJS-12 includes three essential items evaluated in the post-operative time: pain-free daily time, acceptable ROM and hip stability. 

According to the authors, the current literature lacks a psychometric validation of the Italian version of FJS-12 for THA using the COSMIN checklist [22]. A significant validity (except for the preoperative follow-up) and reliability of the Italian translation of FJS-12 employed for THA, compared to WOMAC, was seen. 

According to the article published by Terwee et al. [23], the FJS-12 did not reach a sufficient value of responsiveness. The results of the current study showed an MDC greater than MIC in the preoperative and post-operative follow-ups after 1, 3 and 6 months (Table 2). Therefore, the MDC at every follow-up was greater than the corresponding MIC. For this reason, no positive evaluation for responsiveness could be provided. 

With the exception of the preoperative period, the data showed high internal consistency (Table 2). Supporting these results, the studies of Sethy et al., Hamilton et al. and Klouche [25,26,27] reported a great level of internal consistency for FJS-12 in THA for the Indian, English and French language. The test-retest reliability was assessed after a week, reporting high value for all patients. Several studies also reported a good ICC [25,27,28,29,30]. Since orthopaedic clinical research frequently applies the Italian version of the WOMAC, it serves as a reliable alternative to the FJS-12. Findings from the study supported a high-moderate correlation between the two surveys, with the exception of the time before surgery. These data confirm the results found in the studies of Behrend et al., Klouche et al. and Thompson et al. [16,27,31] on THA. Only a minor association between these scores was found in the article published by Sansone et al. that evaluated the association between the FJS-12 and WOMAC scores. However, this study focused on a different cohort of patients receiving Total Knee Arthroplasty [32]. These data require further investigation since such results could demonstrate a difference between FJS-12 and WOMAC, according to the surgery conducted.

A large ES of FJS-12 was measured preoperatively to 1 month after the procedure (*p* < 0.001). WOMAC reported a significant ES within the same time frame (*p* < 0.001). A high ES was also measured between preoperative time, 3 months, and at the final follow-up (*p* < 0.001).

Currently, new technologies and prosthesis implants assert a more precise score to evaluate postoperative patients’ results [33]. For this reason, it is necessary to design new valid scores for specific procedures, finding tools to distinguish between acceptable and optimal results adequately. The limitation in recognizing a slight difference between acceptable and optimal results is one of the limits of WOMAC. This tool overlooks the high ceiling effect, omitting essential changes in patients during the postoperative period, especially among cases with better outcomes [15,34]. Behrend et al. [16] described no relevant ceiling effect for the FJS-12, and this data was confirmed by other authors [27,29,30,31]. 

The ceiling effect was similar to that which was noted in the initial FJS-12 trial [16]. The floor effect was 0% for both at every follow-up. The floor effect results were similar to the ones found by Behrend et al. (3.3% for FJS-12 and 0.4 for WOMAC) [16] and equal to the results found by Klouche et al. [27].

FJS-12 demonstrated a lower ceiling impact at 6 months and a larger ceiling effect at 1 and 3 months when compared to WOMAC (Table 6). The FJS-12 was more effective in stratifying upper ranges of scores as a result of the exhibited low ceiling and floor effect, which was acceptable. 

On the other hand, the study performed by Bramming et al. [35], on the other hand, detected no relevant floor or ceiling effect, with a high level of responsiveness. However, this study was conducted on patients undergoing arthroscopic hip treatment, differing from the cohort on which this study focused.

The preoperative value of FJS-12 in this study (35.08 ± 15.32) compared to the same value found by the study performed by Hamilton et al. [26] (12.3 ± 15.9) is greater. Even after a follow-up of six months, these findings are still higher. However, cross-cultural variation could have impacted how the research differed [36]. 

To the knowledge of the authors, this is the first research to perform a psychometric validation of the Italian version of FJS-12 for THA. Limited studies evaluated the FJS-12 preoperatively and postoperatively since, as a post-operative measuring device, the FJS-12 was created [26,36]. Collecting preoperative data could provide a reference point for comparing post-operative results. This information may provide more precise knowledge of the benefit of a THA or a joint replacement. 

This study presents some limitations. First, the subjectivity of the questionnaires and their subsequent outcomes lower the experimental equipment’s reliability, in line with the current literature available. This highlights, according to the aim of this study, the need for a coherent language adaptation for the PROMs applied in clinical practice to limit measurement errors. 

As stated by the study of Robinson et al. [37], patients are more likely to experience manageable postoperative symptom states if their baseline function is better. For this reason, the comparison of the different studies in the literature is biased by the inclusion criteria chosen by the different authors. 

The short period of follow-up is yet another flaw in this study. Hamilton et al. [26] found that the most meaningful outcome variations become visible after 1 year, augmenting the effect size. For this reason, further research with longer post-operative follow-ups is encouraged. Additionally, this study did not set out to evaluate the FJS-12′s sensitivity to vary after some time.

## 5. Conclusions

Good test-retest reliability and a mild association with the WOMAC were evaluated for FJS-12. Under the first four months, FJS-12 had a lower ceiling effect than WOMAC. It is recommended to use this score in clinical research concerning the outcomes of THA. FJS-12 could be a useful measure to distinguish between good or exceptional results following THA given the growing amount of THA procedures performed each year and the rising patient expectations.

However, it is suggested to continue with further follow-ups to elongate the study period and permit the analysis of the variation of results according to time. 

## Figures and Tables

**Table 1:** Summary of results of WOMAC.

	FJS-12	WOMAC	FJS-12	WOMAC	FJS-12	WOMAC	FJS-12	WOMAC	FJS-12	WOMAC
	Pre-op		2 Weeks		1 Month		3 Months		6 Months	
**Mean**	35.076	40.587	32.689	19.192	81.326	82.405	80.985	93.826	80.606	94.943
**N**	55	55	55	55	55	55	55	55	55	55
**Std. Deviation**	15.319	18.310	15.177	24.338	18.993	9.609	13.424	5.390	15.825	6.388
**0.5 SD (MIC)**	7.659	9.155	7.588	12.169	9.496	4.805	6.712	2.695	7.912	3.194

**Table 2:** SEM, MDS and MIC of FJS-12 in different follow-ups.

	FJS-12 Pre-op	FJS-12 2 Weeks	FJS-12 1 Month	FJS-12 3 Months	FJS-12 6 Months
**Cronbach’s α**	0.673	0.733	0.922	0.735	0.828
**SEM**	8.760	7.842	5.304	6.911	6.563
**MDC**	24.281	21.737	14.703	19.155	18.192

**Table 3:** Pearson correlation coefficient.

Follow-Up	Pearson Correlation Coefficient	*p*-Value
*Pre-op*	0.287	*p* = 0.002 *
*1 month*	0.702	*p* < 0.001 *
*3 months*	0.516	*p* < 0.001 *
*6 months*	0.585	*p* < 0.001 *

*: *p* < 0.001.

**Table 4:** Responsiveness of FJS-12 over time.

Time		Mean Difference	Std. Error	Sign	95% Confidence Interval for Difference		SD	ES
					Lower Bound	Upper Bound		
***Pre-op***	***1 month***	**−46.25**	**2.461**	<0.001 *	**−51.184**	**−41.316**	**18.252**	2.534
	*3 months*	−45.909	3.177	<0.001 *	−52.278	−39.540	23.559	1.949
	*6 months*	−45.53	3.477	<0.001 *	−52.500	−38.560	25.783	1.766
*1 month*	*3 months*	0.341	3.380	0.920	−6.435	7.117	25.063	0.014
	*6 months*	0.720	3.626	0.843	−6.550	7.989	26.890	0.027
*3 months*	*6 months*	0.379	2.413	0.876	−4.459	5.216	17.895	0.021

*: *p* < 0.001.

**Table 5:** Responsiveness of WOMAC over time.

Time		Mean Difference	Std. Error	Sign	95% Confidence Interval for Difference		SD	ES
					Lower Bound	Upper Bound		
***Pre-op***	***1 month***	**−41.818**	**2.705**	<0.001 *	**−47.241**	**−36.395**	**20.060**	2.085
	*3 months*	−53.239	2.466	<0.001 *	−58.182	−48.295	18.286	2.911
	*6 months*	−54.356	2.494	<0.001 *	−59.357	−49.355	18.498	2.939
*1 month*	*3 months*	−11.42	1.475	<0.001 *	−14.378	−8.463	10.942	1.044
	*6 months*	−12.538	1.479	<0.001 *	−15.504	−9.572	10.971	1.143
*3 months*	*6 months*	−1.117	0.853	0.196	−2.828	0.593	6.326	0.177

*: *p* < 0.001.

**Table 6:** Ceiling and floor effect of FJS-12 and WOMAC.

		Ceiling (%)	Floor (%)
**FJS-12**	Pre-op	0	0
	1 month	25.5	0
	3 months	9.1	0
	6 months	12.7	0
**WOMAC**	Pre-op	0	0
	1 month	0	0
	3 months	5.5	0
	6 months	27.3	0

## Data Availability

The data presented in this study are available on request from the corresponding author. The data are not publicly available due to privacy.
